# Hst1/Gel-MA Scaffold Significantly Promotes the Quality of Osteochondral Regeneration in the Temporomandibular Joint

**DOI:** 10.3390/jfb14100513

**Published:** 2023-10-12

**Authors:** Yiyang Du, Menghan Chen, Jing Jiang, Lei Wang, Gang Wu, Jianying Feng

**Affiliations:** 1 School/Hospital of Stomatology, Zhejiang Chinese Medical University, Hangzhou 310053, China; dudiedie41@163.com (Y.D.); ccchen813@163.com (M.C.); jiang19982016@163.com (J.J.); 2 Wenzhou Institute, University of Chinese Academy of Sciences, Wenzhou 325000, China; wanglei20111213@163.com; 3 Department of Oral Implantology and Prosthetic Dentistry, Academic Centre for Dentistry Amsterdam (ACTA), University of Amsterdam (UvA) and Vrije Universiteit Amsterdam (VU), 1081 LA Amsterdam, The Netherlands; 4 Department of Oral and Maxillofacial Surgery/Pathology, Amsterdam UMC and Academic Center for Dentistry Amsterdam (ACTA), Vrije Universiteit Amsterdam (VU), Amsterdam Movement Science, 1081 LA Amsterdam, The Netherlands

**Keywords:** cartilage, osteochondral, tissue engineering, in vivo

## Abstract

Objective: Our aim was to evaluate the capacity of the human salivary histatin-1-functionalized methacrylic gelatin scaffold to control osteochondral tissue regeneration and repair in vivo in rabbits with major temporomandibular joint dimensional abnormalities. Materials and Methods: In order to compare human salivary histatin-1-functionalized methacrylic gelatin scaffolds to the Blank and Gel-MA hydrogel groups, scaffolds were implanted into osteochondral lesions of a critical size (3 × 3 mm) in the anterior region of the condyle of the temporomandibular joint in New Zealand white rabbits. At 4 weeks after implantation, the repair was evaluated using macroscopic examination, histology, and micro-CT analysis. Results: In the comparison of the composite scaffold group with the Blank and Gel-MA groups, analysis of the healed tissue revealed an improved macroscopic appearance in the composite scaffold group. Regeneration was induced by host cell migration in the Hst1/Gel-MA scaffold group. Conclusions: The current study offers a viable method for in vivo cartilage repair that does not require cell transplantation. Future clinical applications of this strategy’s optimization have many potential advantages.

## 1. Introduction

The mandibular condylar osteochondral complex in the temporomandibular joint (TMJ) plays a paramount role in the growth and articulation of the mandibular bone, contributing to oral movement functions such as chewing and speaking [1]. Osteochondral flaws in the mandibular condyle, which may be caused by recent injuries, excessive amounts of stress, or aberrant immunological reactions [2], may result in lifelong discomfort and restricted jaw motion [3]. The repair of osteochondral defects is difficult due to a poor ability to self-heal. Cartilages bear no vascular structure, which results in the absence of a classic healing cascade and blood-borne multipotent mesenchymal stem cells (MSCs) in cartilage defects [4]. Furthermore, in the surrounding cartilage tissue, the mature chondrocytes have limited ability to migrate and repair [5]. The blood supply from bone tissues may, to some extent, initiate traditional healing processes and attract MSCs [6], but this is not sufficient to enable the full repair of osteochondral lesions and invariably results in the development of fibrous scar tissue [7]. Meanwhile, the reduced area of the condylar head results in abnormal mechanical overloading, which may cause secondary mechanical damage [8]. Osteochondral defects can be managed in the clinic through a variety of autografts, such as autologous chondrocyte implantation [8], mosaicplasty, and stem cells [9], in order to provide regenerative cells. However, their usage is restricted by the scarcity of autografts, donor site discomfort, and morbidity [10]. Consequently, the quantitative and qualitative restoration of osteochondral tissues remains highly challenging. In recent years, tissue engineering (TE) techniques have shown promising potential in applications to repair osteochondral defects [11].

The native TMJ microstructure consists of three distinct structurally and functionally heterogeneous and anisotropic regions: the fibrocartilage, the subchondral bone, and the calcified cartilage. Therefore, in the field of TE, three components with distinct physicochemical and biological properties have become increasingly used in the construction of material scaffolds to aid in the regeneration of bone, cartilage, and the osteochondral interface individually [12]. Such intricate designs are, however, less suited to industrial manufacturing and medical applications. In our recent study, we developed a novel TE construct in which methacrylate gelatin (Gel-MA) hydrogel acts as an osteochondral-conductive scaffold [13] and human salivary histatin-1 (Hst1) functions to recruit and activate regenerative cells [14,15,16,17,18,19,20]. In comparison with zonally structured TE constructs, this Hst1/Gel-MA hydrogel is much less complicated; thus, it has greater application potential. As indicated by the much higher ICRS (International Cartilage Repair Society) score of the Hst1/Gel-MA group compared with the Gel-MA group and Blank group, our data demonstrate that this scaffold helps to completely fill a lesion with a smooth surface and good integration to surrounding tissues. Consistent with the macroscopic observations, histological analysis reveals that Hst1/Gel-MA results in significantly higher levels of collagen II, aggrecan, and collagen fiber expression than the Gel-MA group and Blank group, as well as more newly created subchondral bone and cartilage. These results indicate that Hst1/Gel-MA is efficacious in re-establishing the macroscopic structures and basic compositions of osteochondral tissues [21], suggesting that Hst1/Gel-MA can be used to treat osteochondral lesions in the TMJ, which is a very interesting potential application. However, in order to confirm its application potential, the quality of the newly regenerated osteochondral tissues, through bone microstructure, bone remodeling, neo-vasculation, collagen types, and distributions, remains to be elucidated.

In this study, we adopted micro-computed tomography (micro-CT) analysis, a polarized light microscope, and immunohistochemical staining to analyze the quality of the freshly formed osteochondral tissues induced by Hst1/Gel-MA compared with those induced by the Blank control and Gel-MA.

## 2. Materials and Methods

### 2.1. Preparation of Hst1/Gel-MA Polymer Solution

Based on the previous study, we explored the optimal concentration of this scaffold used in TMJ osteochondral defects in vivo [21]. Freeze-dried Gel-MA macromer (200 mg), purchased from the Wenzhou Institute, was dissolved in 1 mL of phosphate-buffered saline (PBS) containing 0.5% (*w*/*v*) Irgacure 2959 (2-hydroxy-1-(4-(hydroxyethyl)phenyl)-2-methyl-1-propanone, CIBA Chemicals, Basel, Switzerland) at 80 °C. In a water bath that was maintained at a consistent temperature, the prepolymer mixture was kept at 40 °C.

Preoperatively, a 500 μg sterile lyophilized linear Hst1 peptide sample, purchased from the University of Amsterdam (Amsterdam, Holland) and stored at −20 °C, was dissolved in 21.1 μL (equivalent to the volume of the defect) of Gel-MA prepolymer solution to obtain a photopolymerized Hst1/Gel-MA polymer solution. Before use, the prepolymer solution was filtered through a bacterial filter. Prior to surgery, it was freshly prepared and stored in sterile bottles.

### 2.2. Animal Surgery

**Scheme 1:**
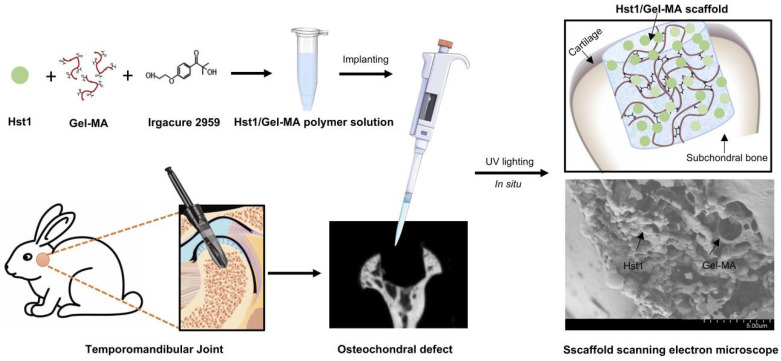
Procedures of Hst1/Gel-MA scaffold implantation in rabbit mandibular condylar osteochondral defect (3 mm × 3 mm).

Adult male New Zealand white rabbits (*n* = 18) were used to construct mandibular condylar osteochondral defect models following a previously described protocol [21]. All surgical procedures were approved by the Zhejiang Chinese Medical University Ethics Committee. Briefly, after general anesthesia, a 3 mm diameter drill was used to create an osteochondral defect, 3 mm in diameter and 3 mm in depth, in the anterior region of the superior surface of the right condylar. Next, three treatments were assigned at random to fill the osteochondral defects: (1) Blank group (*n* = 6): empty defect; (2) Gel-MA group (*n* = 6): a pure Gel-MA polymer solution was embedded in the defect; (3) Hst1/Gel-MA group (n = 6): an Hst1/Gel-MA scaffold polymer solution was embedded in the defect. The polymer solution was crosslinked using ultraviolet radiation (365 nm, 90 s) in situ in the Gel-MA group and Hst1/Gel-MA group. Each animal received unilateral surgery (Scheme 1). 

### 2.3. Analysis of Micro-Computed Tomography (Micro-CT) 

Rabbits were euthanized to collect samples 4 weeks after condyle surgery. The samples (n = 18) were measured with a micro-CT (SkyScan1276, Brooke Company, Germany) scanner. The following scan parameters were chosen: exposure duration was 400 ms, scaled image pixel size was 9.03 μm, voltage was 85 kV, and current was 200 μA. Three-dimensional reconstruction was conducted using NRecon software (V1.7.3.0). After calibration of the scans with the standard body model, a cylindrical region of interest (ROI) (3 mm in diameter and 3 mm in height) was selected in the defect region. By image processing, 2D pictures were taken at the level of the biggest flaw in the coronal tomography scans. Following the manufacturer’s instructions, CTAn software (V1.18.8.0) was used to measure a number of microarchitecture parameters, including bone mineral density (BMD), bone volume/tissue volume (BV/TV), trabecula thickness (Tb.Th), and trabecula spacing (Tb.Sp).

### 2.4. Histological Assessment

The formalin-fixed specimens (n = 18) were decalcified with 10% EDTA (ethylene diamine tetraacetic acid)-buffered saline solution for 2 months. The decalcified samples were incised from the largest sagittal at the center of the defect, dehydrated, and embedded in paraffin blocks. A microtome (Leica RM2265) was used to slice histological sections (6 μm). The prepared sections were stained using hematoxylin and eosin (HE), Safranin-O/Fast Green (SO/FG), Masson, Sirius Red, tartrate-resistant acid phosphatase (TRAP), and alkaline phosphatase (ALP). For each staining, one section per sample was picked for analysis. 

Sections were examined microscopically using scan images captured with a Digital Pathology Slide Scanning System (Hamamatsu, Japan), polarized light microscopy, and digital images. The growth of new tissue, new cartilage, and new bone; the thickness of new cartilage and bone; proteoglycan content; Type I collagen (Col I) fraction of defect; Type II collagen (Col II) fraction of cartilage; and Type III collagen (Col III) fraction of bone were detected and calculated with Image Pro Plus 6.0, a program for image analysis. The areas of new cartilage, new bone, and collagen were selected by researchers. 

**Figure 1:**
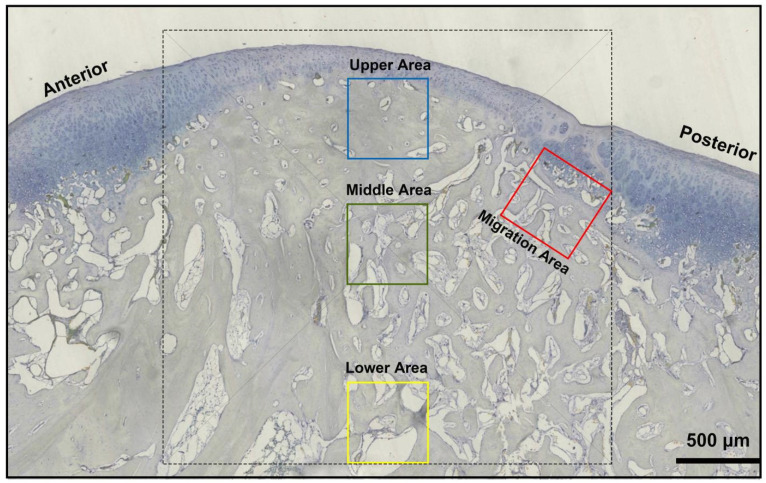
Schematic diagram of subchondral bone division. The red frame is defined as the migration area, located at the lower part of the cartilage at the edge of the defect. The green frame is defined as the middle area, located in the center of the defect. The blue frame is defined as the upper area, located in the upper part of the subchondral bone. The yellow box is defined as the lower area, located at bottom of the subchondral bone defect.

Multinucleated cells that were TRAP-positive were used to define osteoclast activity, and bone cells that were ALP-positive were used to describe osteoblast activity. According to the method shown in Figure 1, four regions of subchondral bone (migration area, upper area, middle area, lower area) were selected for analysis. A subregional count of the TRAP-positive multinuclear cells and the ALP-positive cells in the subchondral bone was performed. 

### 2.5. Immunohistochemistry Analysis

For the purpose of finding possible proteins linked to differentiation, immunohistochemistry was used. Col II, Aggrecan, Platelet endothelial cell adhesion molecule-1 (CD31), and Vascular endothelial growth factor (VEGF) were detected using VEGF (JH121, diluted 1:100, 2 μg/mL), CD31 (c31.7, diluted 1:100, 2 μg/mL), Collagen-II (bs-10589R, diluted 1:100, 2 μg/mL), and Aggrecan (AB1031, diluted 1:100, 2 μg/mL) primary antibodies via immunohistochemical staining, respectively. Integrated optical density, or IOD, was used to express the collagen II and Aggrecan content, while the content of VEGF and CD31 were expressed by positive cell number, measured using Image Pro Plus 6.0.

### 2.6. Statistical Analysis

The mean value and standard deviation (SD) of the results were presented; GraphPad Prism 8 and SPSS 26 statistical software were used to perform statistical analyses. The threshold of statistical significance was established at *α* = 0.05 (*p* < *α*). Statistical analysis was carried out using one-way analysis of variance (ANOVA) and Tukey’s test for multiple comparisons. Statistical significance was denoted by the following symbols: * *p* < 0.05, ** *p* < 0.01, and *** *p* < 0.001.

## 3. Results

### 3.1. Clinical Observation and Micro-CT Analysis

**Figure 2:**
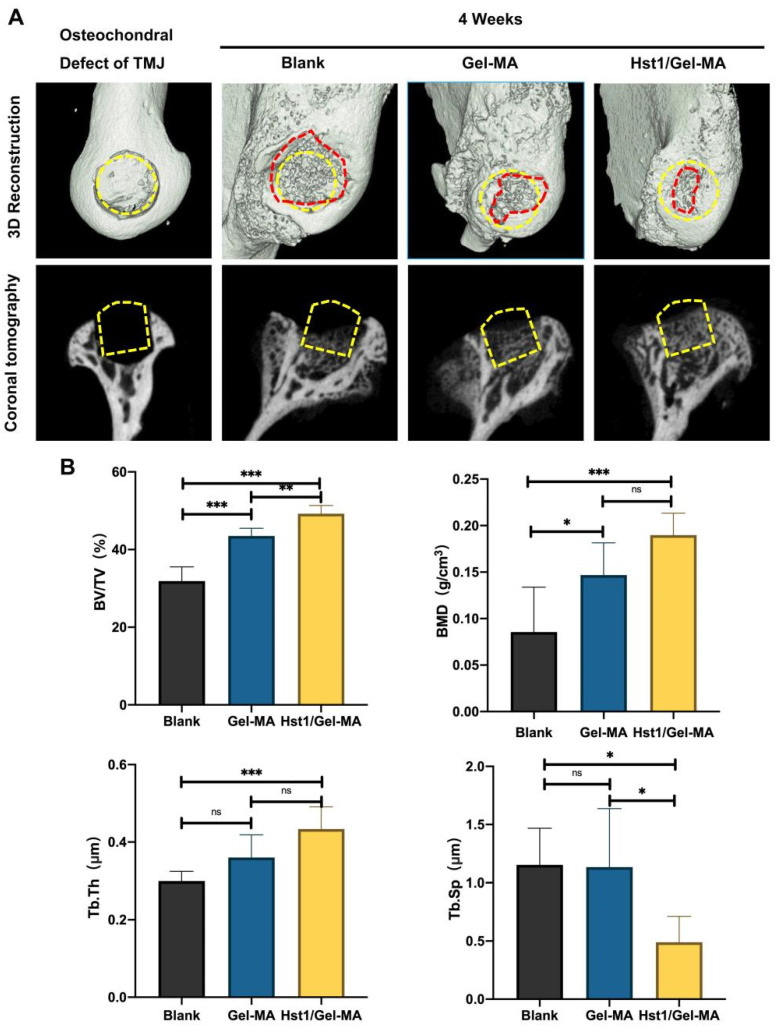
Representative micro-CT images and quantitative morphometric analysis of rabbit mandibular condylar osteochondral defects. (**A**) Three-dimensional reconstruction and representative coronal images of the osteochondral defect model, in which yellow circles and boxes indicate the extent of the original defect and red dashed lines show the extent of the defect at 4 weeks. (**B**) Metrological analysis of bone volume/total volume (BV/TV), trabecular thickness (Tb.Th), trabecular spacing (Tb. Sp), and bone mineral density (BMD). Error bars are mean ± SD (n = 6, * *p* < 0.05, ** *p* < 0.01, and *** *p* < 0.001).

At 4 weeks postoperative, all animals had healed successfully and ate sufficient food to maintain their starting weight up to the time of sacrifice. Representative micro-CT images of each group are shown in Figure 2A. Different patterns of new subchondral bone development were seen in the three groups. Coronal tomography corroborated the results found in the 3D reconstruction: the osteochondral defect of the Hst1/Gel-MA group was almost filled with well-integrated new tissue. A quantitative morphometric analysis (Figure 2B) showed that the BV/TV in the Hst1/Gel-MA group was markedly higher than that in the Gel-MA group and the Blank group (*p* < 0.01, *p* < 0.001, respectively). Additionally, the Hst1/Gel-MA group considerably outperformed the Blank group in terms of Tb.Th and BMD (*p* < 0.001). Meanwhile, between the Gel-MA group and the Hst1/Gel-MA group, there was no statistical difference. Furthermore, compared with the Gel-MA group and the Blank group, the Tb.Sp of the Hst1/Gel-MA group was statistically lower (*p* < 0.05).

### 3.2. Histological Assessment

**Figure 3:**
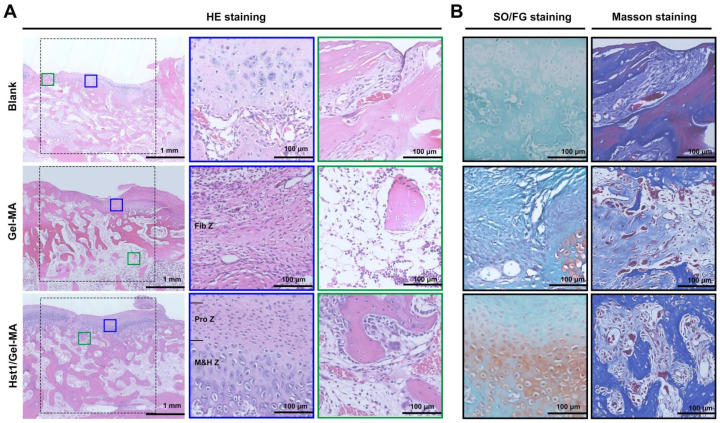
HE, SO/FG, and Masson staining of rabbit mandibular condylar osteochondral defects. (**A**) HE staining. Black dashed boxes show the extent of the original defects (3 mm × 3 mm), blue solid boxes are magnified images of newly formed articular cartilage, and green solid boxes are magnified images of newly formed subchondral bone. (**B**) SO/FG staining of new cartilage center, Masson staining of newly formed subchondral bone.

**Figure 4:**
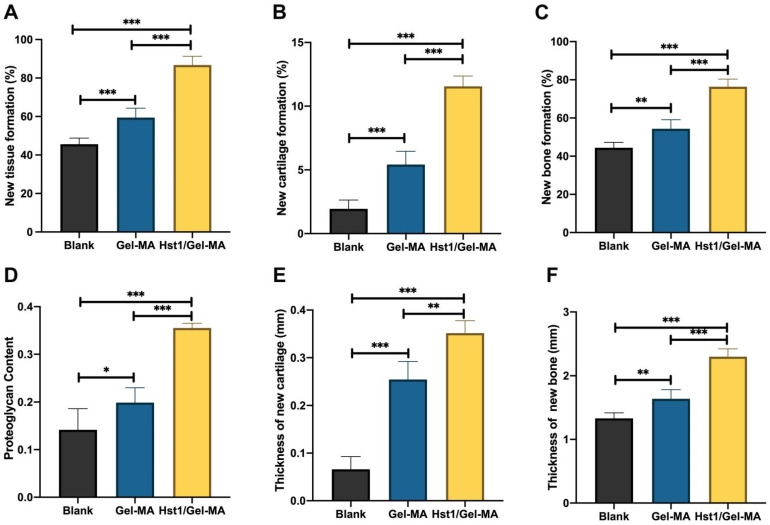
Histological evaluation of rabbit condylar osteochondral defects at 4 weeks postoperative. (**A**) New tissue formation, (**B**) new cartilage formation, (**C**) new bone formation, (**D**) evaluation of proteoglycans in the cartilage matrix, (**E**) thickness of new cartilage, (**F**) thickness of new bone. Error bars are mean ± SD (n = 6, * *p* < 0.05, ** *p* < 0.01, and *** *p* < 0.001).

A notable finding in the Hst1/Gel-MA group was the simultaneous regeneration of articular cartilage and subchondral bone that was clearly detectable, as shown in Figure 3A. The proportion of new cartilage and subchondral bone as well as the mean thickness of new cartilage and subchondral bone were significantly higher in the Hst1/Gel-MA group than in the other two groups (Figure 4). 

In the Hst1/Gel-MA group, the integrated reparative cartilage tissue showed typical columnar aligned chondrocytes that were stratified into fibrous, proliferative, mature, and hypertrophic zones (Figure 3A). The Hst1/Gel-MA group had a noticeably increased amount of proteoglycan deposition (Figure 3B and Figure 4D). The vascularized subchondral bone had both osteoblasts and osteoclasts in significant numbers (Figure 3B). Evidence of a good transition between the cartilage layer and the subchondral bone area was detected. In comparison, sufficient osteochondral defect repair was not observed in the Gel-MA group. Widespread fibrous tissues filled the defect without a fibrocartilage structure. Meanwhile, the Blank group essentially remained in an unrepaired state, with irregular immature cartilage negatively stained by SO/FG and exposed subchondral bone. 

**Figure 5:**
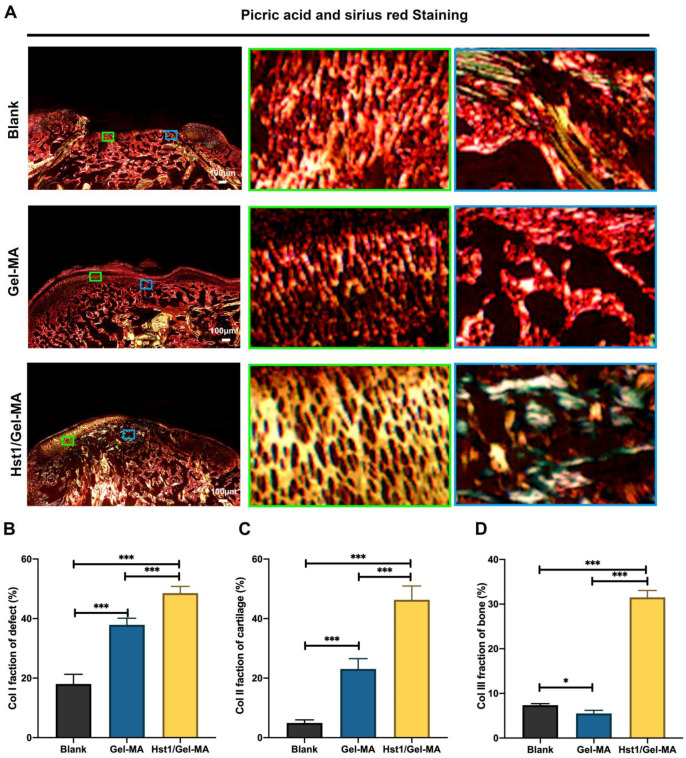
Polarized light microscopy images of Sirius red-stained condylar osteochondral defects at 4 weeks postoperative. (**A**) Polarized light Sirius red staining microscopy. The solid green box shows the distribution of Col II (colorful reticular distribution) in the new cartilage and the solid blue box shows the distribution of Col III (greenish) in the new subchondral bone. (**B**) Metrological analysis of the Col I fraction of defect. (**C**) Metrological analysis of the Col II fraction of cartilage. (**D**) Metrological analysis of the Col III fraction of the subchondral bone. Error bars are mean ± SD (n = 6, * *p* < 0.05, and *** *p* < 0.001).

The prominent collagen matrix formed in the developing tissues was further seen using polarized light Sirius red staining microscopy (Figure 5A). Most Col I organization was observed in the repaired tissues in the Hst1/Gel-MA group. In the Hst1/Gel-MA group, the neo-cartilage was rich in Col II, with a regular multicolored fiber network arranged closely and a neo-bone with noticeably large amounts of Col III. A rough surface and damaged, disorganized collagen fibers were present in the Blank group’s defects. Additionally, the Hst1/Gel-MA group had considerably higher proportions of Col I in the defect, Col II in the cartilage, and Col III in the bone than the other two groups (*p* < 0.001, Figure 5B). 

### 3.3. IHC Analysis

**Figure 6:**
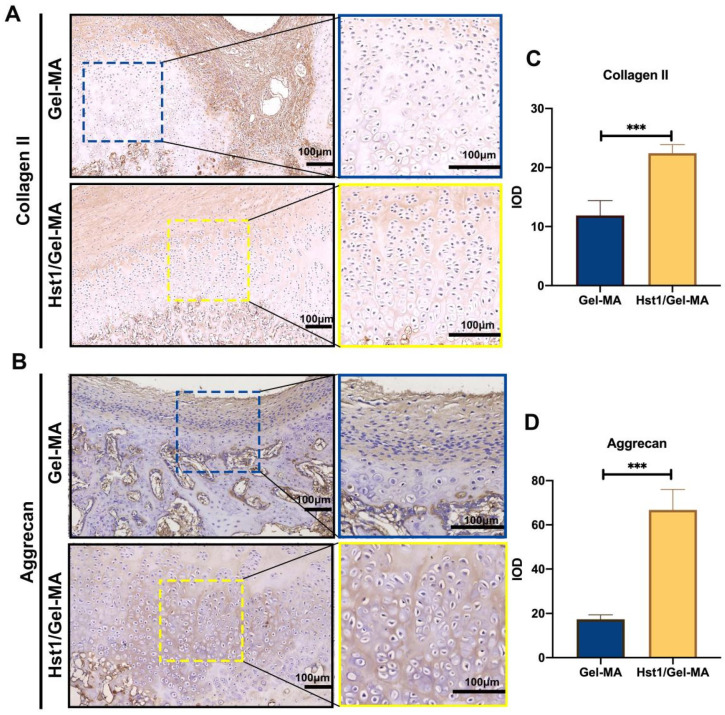
Immunohistochemical micrographs and quantitative analysis of cartilage matrix markers Col II and aggrecan were performed in the Gel-MA and Hst1/Gel-MA groups at 4 weeks postoperative. (**A**) Immunohistochemical micrographs of collagen II. (**B**) Immunohistochemical micrographs of aggrecan. (**C**) Expression of collagen II at 4 weeks. (**D**) Expression of aggrecan at 4 weeks. Error bars are mean ± SD (n = 6, *** *p* < 0.001).

In the Hst1/Gel-MA group, Col II and aggrecan deposition in cartilage tissue reached that of normal cartilage, but minimal Col II and aggrecan staining was evident in the Gel-MA group (*p* < 0.001, Figure 6). The cartilage layer in the Blank group had an unorganized distribution of chondrocytes and little expression of Col II or aggrecan. The outcomes of HE staining, SO/FG staining, and Sirius red staining agreed with those of IHC.

**Figure 7:**
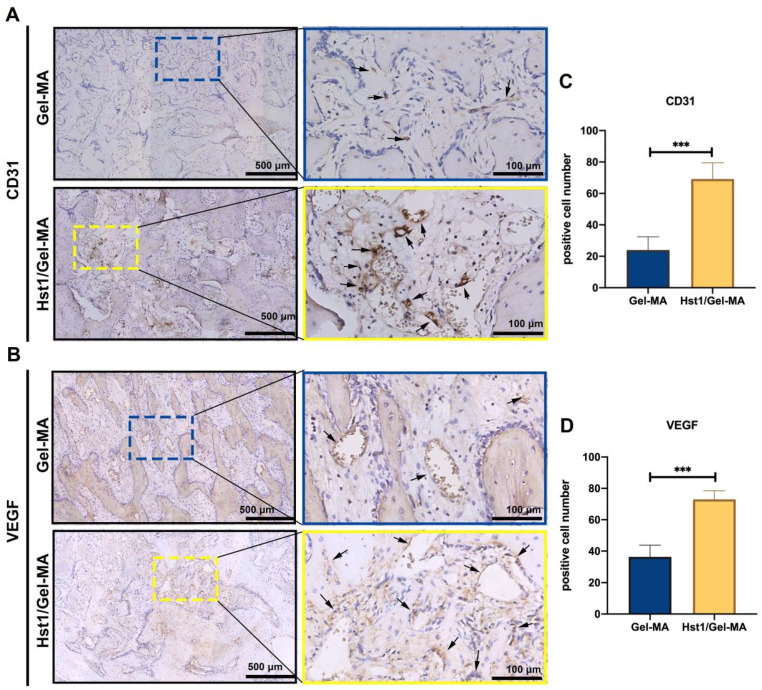
Immunohistochemical micrographs and quantitative analysis of angiogenic markers CD31 and VEGF were performed in the Gel-MA and Hst1/Gel-MA groups at 4 weeks postoperative. Black arrows indicate positively stained vessels. (**A**) Immunohistochemical micrographs of CD31. (**B**) Immunohistochemical micrographs of VEGF. (**C**) Expression of CD31 at 4 weeks. (**D**) Expression of VEGF at 4 weeks. Error bars are mean ± SD (n = 6, and *** *p* < 0.001).

The expression of angiogenic markers including CD31 and VEGF was measured using IHC to investigate the angiogenic effect of the Hst1/Gel-MA scaffold (Figure 7). The subchondral bone of the Hst1/Gel-MA group had higher levels of CD31 and VEGF expression than that of the Gel-MA group, and these findings were compatible with Masson staining (*p* < 0.001). 

### 3.4. Assessment of Bone Metabolism Activity in Subchondral Bone

**Figure 8:**
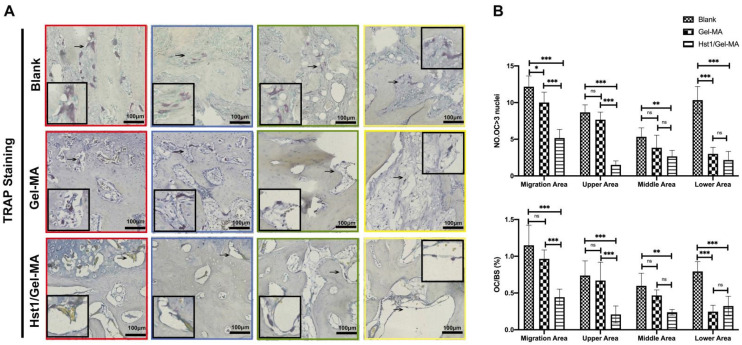
TRAP staining of micrographs and quantitative analysis of osteoclasts were performed in mandibular condylar subchondral bone at 4 weeks postoperative. Red boxes represent areas of cartilage migration. Blue boxes represent the upper part of the defect centre. Green boxes represent the middle part of the defect centre. Yellow boxes represent the lower part of the defect centre. Black arrows indicate positive staining areas. (**A**) TRAP staining of micrographs of the subchondral bone. (**B**) Metrological analysis of osteoclasts. Error bars are mean ± SD (n = 6, * *p* < 0.05, ** *p* < 0.01, and *** *p* < 0.001).

**Figure 9:**
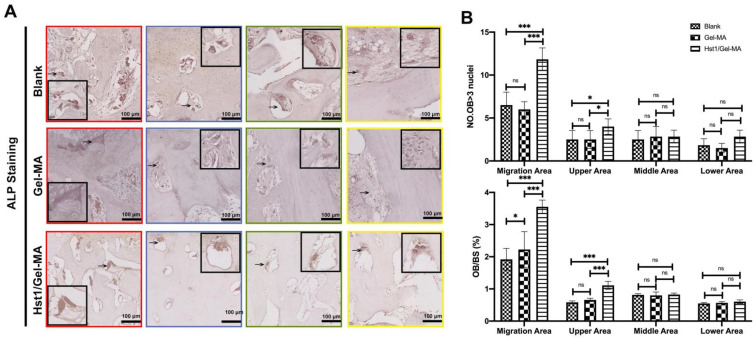
ALP staining of micrographs and quantitative analysis of osteoblasts were performed for mandibular condylar subchondral bone at 4 weeks postoperative. Red boxes represent areas of cartilage migration. Blue boxes represent the upper part of the defect centre. Green boxes represent the middle part of the defect centre. Yellow boxes represent the lower part of the defect centre. Black arrows indicate positive staining areas. (**A**) ALP staining of micrographs of the subchondral bone. (**B**) Metrological analysis of osteoblasts. Error bars are mean ± SD (n = 6, * *p* < 0.05, *** *p* < 0.001).

To more accurately evaluate the bone metabolism’s kinetics, we observed the migration area, upper area, middle area, and lower area after TRAP/ALP staining (Figure 8 and Figure 9). The number of TRAP-positive multinuclear cells and ALP-position multinuclear cells in each region was not uniform throughout the structure. In all four locations, the osteoclast count was considerably lower for the Hst1/Gel-MA group than for the Blank group (*p* < 0.001), and in the migratory area and upper area, the Hst1/Gel-MA group had more osteoblasts than the Blank group (*p* < 0.001). Meanwhile, the number of osteoclasts in the migratory and upper area of the Hst1/Gel-MA group was much lower than in the Gel-MA group (*p* < 0.01), and the quantity of osteoblasts was significantly larger than that of the Gel-MA group (*p* < 0.05), despite intragroup variability in the middle and lower area. 

## 4. Discussion

The remodeling of osteochondral defects is extremely difficult with self-healing due to the continuous mechanical loading, inadequate avascularity of the cartilage tissue, as well as deficiencies in MSC migration and proliferation [5]. In this study, we created a nonlayered Hst1-functionalized Gel-MA scaffold and implanted it into critical-size, weight-bearing osteochondral lesions in the rabbit mandibular condylar to provide a viable therapy alternative. We found that the Hst1/Gel-MA group was an efficacious construct for simultaneously assisting in the regeneration of fibrocartilage and bone compared with the other two groups. 

The quality and quantity of freshly formed subchondral bone in the Hst1/Gel-MA group were considerably superior to those of the other two groups. The 3D reconstruction images in this study showed that the contours of the condylar surface gradually recovered; however, poor bone healing was observed in the center of the defect. This demonstrated that new bone formed gradually from the defect edge to the defect center as the scaffold deteriorated. Histological observations of the sections showed that the Hst1/Gel-MA scaffold promoted regeneration and extracellular matrix mineralization of the new bone. The Micro-CT data demonstrated that the Tb.Sp, BV/TV, Tb.Th, and BMD values decreased in the Hst1/Gel-MA group, along with improvements in bone structural characteristics. Histological staining analysis confirmed the Hst1/Gel-MA scaffold boosted subchondral bone repair, generating thicker bone. The images of Sirius red staining under polarized light showed that Hst1/Gel-MA had the highest proportion of Col I in the newly formed tissue and the highest proportion of Col III in the new subchondral bone, confirming that the bone tissue was properly restored. Col I binds to the mineralized collagen fibrils already present in the bone, controlling the creation of sacrificial bone and the nucleation and growth of bone mineral crystals to further increase bone toughness [22], thus endowing the tissue with load-bearing mechanical properties [23]. Col III is a crucial regulator of collagen fiber structure and the biomechanics of articular cartilage [24], giving tissues tensile strength and integrity [25]. Col III acts as a modifier of the fibrillar network during tissue healing [26], promoting healing. Moreover, the new bone was integrated with the adjacent host tissue well, without obvious cracks or empty cavities, indicating that the Hst1/Gel-MA scaffold’s ability to promote bone healing was superior to that of the other two groups. However, collagen type X is a marker of cartilage proliferation for the formation of calcified cartilage, and the expression or non-expression of collagen type X in chondrocytes is an important indicator of the biological traits of chondrocytes. We have conducted studies on collagen types I, II, and III and need to refine our analysis of collagen type X. 

Gel-MA scaffolds are a beneficial component for bone healing, providing support for bone matrix formation and mineralization [27]. In this study, we evaluated the repair via histology and micro-CT analysis, and we also need to perform SEM analysis of the calcium phosphate deposits in the scaffolds to clarify the mineralization. Gel-MA gels rapidly in situ, making it easily applicable to defects and confirming it can assist in the creation of three-dimensional tissues and cell proliferation [28]. In our study, the Hst1/Gel-MA scaffold polymer solution was photopolymerized using ultraviolet rays (365 nm, 90 s) in situ. UV light has been widely used in light crosslinking. The concern about its biosafety is mainly due to its phototoxicity to living cells, which does not affect the properties of Hst1 [29]. It has also been shown that Hst1 can rapidly target and activate mitochondria after uptake, and it has a protective effect on human epithelial cell damage induced by ultraviolet rays [30]. Gel-MA has numerous benefits, including high biocompatibility, biodegradability, physicochemical tailorability, and affordability [31]. Previously, due to its appropriate biological features and adaptable physical characteristics, it has been employed as a flexible matrix for bone tissue engineering scaffolds [27]. Liang Chen [32] demonstrated that grafting mesoporous bioactive glass nanoparticles into Gel-MA increased the mechanical qualities as well as improved angiogenesis and osteogenesis. Simultaneous mineralization and angiogenesis was effectively achieved using a Gel-MA-based scaffold entrapped with both osteogenic and angiogenic cells [33]. In our study, there was a dynamic balance between scaffold degradation and tissue formation in both the Gel-MA group and the Hst1/Gel-MA group. Our findings revealed that the application of Gel-MA individually resulted in better subchondral bone repair than the Blank group, with greater osteoblast infiltration and a higher subchondral bone mineral content (*p* < 0.05). 

The synergistic osteogenesis and angiogenesis of Hst1 is a potential mechanism for the effective repair of subchondral bone. Hst1 is a salivary bioactive peptide that can stimulate numerous cell activities [14], such as promoting cell adhesion [16] and migration [17], including osteoblasts [18], fibroblasts [19], and adipocytes [20]. The intimate temporal and spatial connection between osteogenesis and angiogenesis is called “angiogenesis–osteogenesis coupling” [34]. Effective bone tissue repair requires a functional vascular structure, which is a prerequisite for bone regeneration [35]. VEGF is a potent angiogenic agent that may boost the migration, proliferation, and survival of vascular endothelial cells [36]. It is necessary for the effective coupling of angiogenesis and osteogenesis during the growth of the skeleton and the repair of broken bones [37]. CD31 exists in vascular endothelial cells and has dual properties, promoting vascular protection and neovascularization. It is believed to play a significant role in the improved regulation of bone development. Immunohistochemical analysis of angiogenic markers in new bone showed that the Hst1/Gel-MA scaffold could significantly enhance the expression of VEGF, which could account for some of the improvement in the vascularized subchondral bone formation (*p* < 0.001). The pro-angiogenesis effect of Hst1 has been widely confirmed [30]. It was recently shown to act as a proangiogenic factor that could activate Rac1 via a signaling pathway, the so-called “RIN2/Rab5/Rac1” axis [15]. This pathway is relevant for angiogenesis and vascular morphogenesis. Other in vivo experiments have also demonstrated that Hst1 alone can promote angiogenesis as required for skin wound healing [38]. Hst1 can significantly promote the expression of a series of vascular markers and enhance the formation of new bone [39]. In addition, Hst1 is a new osteogenic factor, and the uptake of Hst1 by osteoblasts has also been demonstrated [14]. It promotes the cell adhesion, diffusion, and migration of preosteoblasts in vitro, increasing the activity of ALP and encouraging mineralization by promoting the expression of osteogenic genes such as osteocalcin, osteopenia, and Runx2 [40]. 

The Hst1/Gel-MA scaffold can provide a strong bone matrix frame for osteochondral defect healing and provide the possibility for the enhanced proliferation and repair of surface chondrocytes. It is well acknowledged that MSCs, which can move locally to sites of the osteochondral defect and differentiate as needed to replace wounded tissue, are responsible for osteochondral defect rehabilitation [41]. Further, the extent and outcome of the repair and remodeling responses of osteochondral defects are critically dependent on the reconstruction of subchondral tissue [6]. The condyle is supported by the subchondral bone matrix, which also acts as a stent for cartilage healing. Subchondral bone dynamically adjusts bone density patterns, mechanical properties, trabecular orientation, and proportional parameters; thus, the articular bone contour is preserved, and articular chondrocytes can grow and differentiate in a biomechanically favorable environment [42]. Moreover, the subchondral bone’s vascular system offers limited regenerative capabilities, delivering nutrients to the chondrocytes [43,44]. Studies have shown a linear relationship between cartilage thickness and subchondral bone plate thickness, as well as between the cartilage loss modulus and the BMD of the subchondral bone [45]. 

Based on the excellent subchondral bone healing, the Hst1/Gel-MA group led to better regeneration of cartilage. The chondrocyte architecture and distribution in the new cartilage in the Hst1/Gel-MA group resembled the cartilage tissue around it, including the characteristic fibrous, proliferative, mature, and hypertrophic zones [46]. Chondrocytes are layered and orderly, which helps the cartilage resist continuous pressure. The proliferative zone is home to MSCs that repopulate fibroblasts and chondrocytes, the mature and hypertrophic zones contain embedded mature chondrocytes, and the fibrous zone is primarily composed of fibroblasts [47]. Consistent with our previous findings [21], the cartilage matrix (Col I, Col II, aggrecan, and proteoglycan) was remodeled to the greatest extent in the Hst1/Gel-MA group. The normal functioning of load-bearing cartilage hinges on the composition and structural integrity of the cartilage matrix [48]. The main structural protein in cartilage, Col II, is essential for controlling the mechanical transduction of chondrocytes [49]. Col I, also known as the fibril-forming collagen [50], was found in high concentrations in the fibrous and proliferative zones of TMJ fibrocartilage and subchondral bone. More regular and vertically organized collagens were seen in the newly created cartilage of the Hst1/Gel-MA group, which increased the compression resistance of the cartilage and provided a favorable mechanical environment for chondrocyte proliferation and differentiation. We believe that the Hst1/Gel-MA scaffold aids in the regeneration and repair of the load-bearing osteochondral structure. 

Overall, this study offers in vivo proof-of-concept for using this nonlayered scaffold for the repair of mandibular condylar osteochondral defects. The findings convincingly demonstrate the capabilities of this biomimetic scaffold to assist and guide the host to higher quality healing. Complete tideline structures could not be observed at the osteochondral interface of the three groups; this tideline indicates the limiting line for calcification and vascularization, and plays a significant role in preventing the vascular invasion of the cartilage and maintaining a non-mineralized avascular cartilage layer [51]. In such a scenario, direct bone-on-cartilage contact between an opposing host articular surface and an ossification within a dynamic joint may result in chondral injury and an early stage of degenerative degeneration [52]. Hence, the follow-up time should be extended to observe the recovery of the tideline.

## 5. Conclusions

In this study, we designed an unstratified scaffold by integrating Hst1 and Gel-MA for the repair of condylar osteochondral defects. In an experiment in the rabbit condyle with critical-sized osteochondral defects, we found that Hst1/Gel-MA was an efficacious construct that facilitated osteochondral defect repair compared with the Gel-MA group and the Blank group. It is suggested that the Hst1/Gel-MA scaffold might effectively promote the formation of bone and fibrocartilage, both of which had structures similar to that of the host tissue. From this study, it can be concluded that the Hst1/Gel-MA scaffold can be used to repair condylar cartilage defects in vivo and is an attractive candidate scaffold for potential use in clinical applications. To obtain improved osteochondral implants, future research should focus on the scaffold’s fabrication procedure.

## Data Availability

No new data were created or analyzed in this study. Data sharing is not applicable to this article.
